# Relationship between primary eradication of *Helicobacter pylori* and drinking habits in women: collaborative research between a pharmacy and a clinic

**DOI:** 10.1017/S0950268819001730

**Published:** 2019-10-22

**Authors:** Kayoko Ozeki, Michio Asano, Takahisa Furuta, Toshiyuki Ojima

**Affiliations:** 1 Department of Community Health and Preventive Medicine, Hamamatsu University School of Medicine, 1-20-1 Handayama, Higashiku, Hamamatsu, Shizuoka 431-3192, Japan; 2 Asano Clinic, 1628-1 Takatsukacho, Minamiku, Hamamatsu, Shizuoka 432-8065, Japan; 3 Center for Clinical Research, Hamamatsu University School of Medicine, 1-20-1 Handayama, Higashiku, Hamamatsu, Shizuoka 431-3192, Japan

**Keywords:** Drinking habits, first-line eradication, *Helicobacter pylori*, women

## Abstract

*Helicobacter pylori* is a cause of stomach cancer and peptic ulcer. For prevention, improving the eradication rate of *H. pylori* is crucial. However, the association between eradication and lifestyle of infected patients, including alcohol consumption, remains unclear. We explored associations between failed primary eradication therapy and drinking status by sex. This study involved 356 patients who visited a pharmacy with prescriptions for primary *H. pylori* eradication therapy. We assessed drinking habits using a questionnaire. Data on patients with failed primary eradication were provided by the nearby local clinic. We performed logistic regression analysis to examine the effect of drinking habit and frequency of drinking on failed primary eradication by sex. The odds ratio of primary eradication failure in female patients with a drinking habit was 3.75 (*P* = 0.001), but that in male patients was not significant. The odds ratio tended to increase in relation to drinking frequency in women. Frequent consumption of alcohol is not only likely to affect eradication, but also has a large impact on the bodies of women, who are more susceptible than men to the effects of alcohol. Thus, women should take greater care in alcohol consumption.

## Introduction

Pharmacists are indispensable in the medical field in their service to the local community. The concept of the ‘seven-star pharmacist’ where a pharmacist is described as a caregiver, communicator, decision-maker, teacher, lifelong learner, leader and manager was introduced by the World Health Organization (WHO) and adopted by the International Pharmaceutical Federation (FIP) in 2000 [1]. However, in 2006 [2], the function of researcher has been added, because it is essential for excellent pharmacists to conduct research activities.

To date, pharmacists have produced good research results, such as the Asheville Project [3] and the PINCER intervention Project [4], which reported outcomes of community pharmacy research. Results generated from a large-scale study by pharmacists could have a major impact. However, research performed by pharmacists at a local pharmacy in collaboration with a medical institution could reveal lifestyle changes that could facilitate disease prevention and improve quality of life.

In Japan, the need for collaborative research on the pharmacist's role in a pharmacy is recognised [5], but few such studies have been conducted. Under such circumstances, we planned collaborative research between a pharmacy and a local gastroenterology clinic. In conducting the study, we focused on the eradication of *Helicobacter pylori*, which is a cause of stomach cancer and peptic ulcer.

Improvement in the eradication rate of *H. pylori*, a cause of gastric cancer and gastric ulcer, is one of the most important factors in disease prevention [6–9]. Eradication of *H*. *pylori* is reported to protect against progression of premalignant gastric lesions [10], inhibit of peptic ulcer recurrence [11] and reduce the risk of gastric cancer [12]. The primary eradication rate by conventional combination therapy with amoxicillin, clarithromycin and a proton pump inhibitor (PPI) in Japan is reported to be 65.3%, but a study has shown that this rate decreased significantly over the 10 years between 2000 and 2009 [13]. Another study in 2007–2008 has shown a slightly higher value of 74.8% eradication rate [14], although neither value is satisfactory.

Potassium-competitive acid blockers are now commonly used in place of PPIs in *H. pylori* eradication, and have resulted in an improved eradication rate [15]. Nevertheless, it is still important to identify factors contributing to the difficulties in *H. pylori* eradication.

To consider a possible reason for the decrease in the eradication rate, we investigated possible effects of patients’ lifestyle, focusing on the effects of drinking. Several reports have discussed the effects of drinking and eradication [16–18]. However, results are inconsistent and the relationship is yet unknown. Furthermore, very few studies have evaluated the frequency of drinking in the setting of *H. pylori* infection. We therefore conducted stratified analysis using sex or frequency of drinking as parameters, to study the relationship between eradication status and drinking in patients taking primary eradication drugs for *H. pylori* in detail.

## Methods

### Study population and survey procedure

Questionnaire data were collected from 356 patients who visited a pharmacy adjacent to a gastroenterology clinic between April 2013 and February 2014 with a prescription for drugs for primary eradication for *H. pylori* (daily dose of 60 mg lansoprazole, 1500 mg amoxicillin and 800 mg clarithromycin) and then were then analysed. Patients were obliged to complete a questionnaire at their first visit to the pharmacy, meaning that it was possible to obtain data on patient attributes. Investigated attributes were sex, age, drinking status, frequency of drinking per week, smoking status and hay fever status. Exclusion criteria were non-response to these required attributes.

Two months after completing the 1-week course of primary eradication drugs, patients revisited the gastroenterology clinic for assessment of eradication status. The ^13^C-urea breath test was mainly used to assess eradication, and values greater than the cut-off of 2.5‰ were taken to denote failed eradication. Endoscopic examinations together with the rapid urease test were generally carried out in patients 1 year after eradication.

The study was conducted with approval by the ethics committee of Hamamatsu University School of Medicine (No. 14-383). The purpose of the study was explained in writing, and we regarded completion of the questionnaire to indicate consent to participate.

### Statistical analysis

Comparisons in the patients’ drinking status were performed using the chi-square test (Table 1). Because there are large differences in drinking habits between men and women, logistic regression analysis was performed by stratifying data into successful eradication and failure of eradication separately for men and women, to find out the extent to which drinking affects eradication (Table 2). In Model 1, drinking status was the independent variable. An individual who drinks more than once a week was defined as having a drinking habit. In Model 2, age, sex, smoking status and hay fever status were covariates. Hay fever status was used as a covariate, as our previous study showed that eradication tended to be difficult in patients with fay fever [19]. Next, differences in eradication rates depending on the frequency of drinking per week in men and women were analysed (Table 3). The method used for determining frequency of drinking was in accordance with the National Health and Nutrition Survey in Japan, 2015. Furthermore, to identify the association between eradication outcome and differences in frequency of drinking per week, logistic regression analysis was performed by stratifying data for sex and frequency of drinking (no drinking (reference), 1–2 times, 3–4 times, 5–7 times a week) (Table 4). In Model 2, age, sex, smoking status and hay fever status were added as the covariates. SPSS 20 software was used for the statistical analysis.

**Table 1.** Demographic characteristics according to drinking status

Factor	Drinking (−)	Drinking (+)	*P*-value
	*n*/*N* (%)	*n*/*N* (%)	
Sex			
Male	51/209 (24.4)	96/131 (73.3)	<0.0001
Female	158/209 (75.6)	35/131 (26.7)	
Age			
⩽50 years	24/209 (11.5)	14/131 (10.7)	0.600
51–60 years	38/209 (18.2)	26/131 (19.8)	
61–70 years	60/209 (28.7)	45/131 (34.4)	
⩾70 years	87/209 (41.6)	46/131 (35.1)	
Smoking status			
Smoking (−)	200/208 (96.2)	113/129 (87.6)	0.003
Smoking (+)	8/208 (3.8)	16/129 (12.4)	
Hay fever status			
Hay fever (−)	172/209 (82.3)	102/129 (79.1)	0.477
Hay fever (+)	37/209 (17.7)	27/129(20.9)	

*N*, total number of patients who received eradication therapy; *n*, number of patients in each category.

**Table 2.** Association between failure of *Helicobacter pylori* eradication and drinking

		Model 1				Model 2			
Sex	Drinking status	*N*	Univariate analysis			*N*	Multivariate analysis^a^		
			OR	95% CI	*P*-value		OR	95% CI	*P*-value
Male	Drinking (−)	48	1 (Reference)		0.926	48	1 (reference)		0.889
	Drinking (+)	88	0.96	0.44–2.09		87	0.94	0.43–2.09	
Female	Drinking (−)	146	1 (Reference)		**0.001**	145	1 (reference)		**0.003**
	Drinking (+)	35	**3.81**	1.71–8.48		34	**3.75**	1.57–8.94	

*N*, number of patients who received eradication treatment; OR, odds ratio; CI, confidence interval.

a Multivariate analysis was adjusted for age, smoking status and hay fever status.

**Table 3.** Eradication rate without drinking and with drinking (frequency of drinking)

	Frequency of drinking (per week)	*n*/*N* (%)
Male	0	34/48 (70.8)
	1–2	8/14 (57.1)
	3–4	6/9 (66.6)
	5–6	16/19 (84.2)
	7	22/29 (75.9)
	Unknown	11/17 (64.7)
Female	0	122/146 (83.6)
	1–2	8/11 (72.7)
	3–4	3/7 (42.9)
	5–6	2/5 (40.0)
	7	0/3 (0.0)
	Unknown	7/9 (77.8)

*N*, number of patients who received eradication therapy; *n*, number of patients with successful *H. pylori* eradication.

**Table 4.** Association between failure of *H. pylori* eradication and frequency of drinking (per week)

		Model 1				Model 2			
Sex	Frequency of drinking (per week)	*N*	Univariate analysis			*N*	Multivariate analysis^a^		
			OR	95% CI	*P*-value		OR	95% CI	*P*-value
Male	0	48	1 (reference)			48	1 (reference)		
	1–2	14	1.82	0.53–6.21	0.339	14	1.72	0.48–6.19	0.407
	3–4	9	1.21	0.27–5.55	0.802	9	1.20	0.25–5.80	0.822
	5–7	48	0.64	0.25–1.63	0.348	48	0.59	0.23–1.56	0.290
Female	0	146	1 (reference)			145	1 (reference)		
	1–2	11	1.91	0.47–7.71	0.365	11	2.22	0.51–9.61	0.287
	3–4	7	6.78	1.43–32.24	**0.016**	7	**8.48**	1.63–44.23	0.011
	5–7	8	15.25	2.90–80.13	**0.001**	8	**18.13**	2.97–110.58	0.002

*N*, number of patients who received eradication therapy; OR, odds ratio; CI, confidence interval.

a Multivariate analysis was adjusted for age, smoking status and hay fever status.

## Results

Demographic characteristics of patients according to their drinking status are shown in Table 1. Detailed breakdown of patients’ age were two patients in their 20s, 10 patients in their 30s, 24 patients in their 40s, 65 patients in their 50s, 108 patients in their 60s, 118 patients in their 70s and 31 patients in their 80s. There was a significant difference in the proportion of drinkers *vs.* non-drinkers between men and women; 65.3% of men and 18.9% of women were drinkers. Furthermore, the percentage of smokers was significantly high among those who were drinkers. No significant differences were noted between the drinking and non-drinking groups in terms of age or hay fever status.

Results of logistic regression analysis on drinking status and eradication status for each sex are shown in Table 2. In both Models 1 and 2, women who drank were found to be more resistant to eradication. There were no significant relationships observed in men.

Relationships between the eradication rate and frequency of drinking per week are shown in Table 3 for men and women. In men, no relationship between these parameters was found. However, the eradication rate was found to decrease in relation to increasing frequency of drinking per week in women, revealed by the Mantel–Haenszel test for trend (*P* = 0.028).

Results of logistic regression analysis on drinking frequency and eradication status for each sex are shown in Table 4. In both Models 1 and 2, women who drank more than three times a week were found to be more resistant to eradication. The odds ratio tended to increase in relation to drinking frequency. There were no significant relationships observed in men.

## Discussion

To our knowledge, this is the first study in which a relationship was demonstrated between *H. pylori* eradication and patient lifestyles in terms of drinking habit or frequency of drinking per week. This study revealed that eradication failed more frequently in women with a habit of drinking 5–7 days a week; the adjusted odds ratio was 18.13. There have been numerous studies on the association between *H. pylori* infection and alcohol, some of which report an inverse relationship between *H. pylori* infection and alcohol consumption [20, 21]. However, few studies have examined the association between *H. pylori* eradication and alcohol, particularly in terms of sex.

It is well known from previous studies that gastric acid secretion is activated by alcohol consumption [22–25]. Decrease in stomach pH due to gastric acid secretion is known to enhance decomposition of antibiotics, attenuating their efficacy [26]. However, the relationship between drinking habits prior to receiving eradication treatment has not been discussed extensively. This study was conducted to find out the influence of drinking habits established before starting eradication treatment on the rate of success in eradication.

Interestingly, no relationship between drinking habits and failure in eradication was found in men, but a relationship was observed in women. In addition, a dose–response relationship, which is evidence strongly suggesting the relationship between frequency of drinking and eradication, was observed in women, as shown by a significant increase in the odds ratio.

A previous study has shown that eradication using omeprazole, clarithromycin and tinidazole failed more frequently in women than in men [27]. Clarithromycin, an antibiotic contained in the eradication agent, is known to be metabolised mainly by CYP3A4, and there is a likely difference in CYP3A4 activity between men and women [28, 29]. The metabolic clearance rate of CYP3A4 was also found to be higher in women compared with men [30, 31]; in particular, higher clearance rates of erythromycin, a macrolide antibiotic similar to clarithromycin, were seen in women [32]. Therefore, sex differences in CYP3A4 activity and metabolic clearance are likely to directly affect the sex differences in the eradication rate.

Induction in cytochrome P450 including CYP3A4 is known to occur in response to regular, heavy alcohol consumption [33]. This induction of CYP3A4 by alcohol may lead to increased metabolism of clarithromycin, leading to decreased efficacy and failure of eradication in women. Besides, CYP2C19 is also known for enzyme induction in alcohol consumption [34]. Regarding CYP2C19 genotypes, rapid and intermediate metabolisers of PPIs reportedly have a lower eradication rate compared to poor metabolisers [35]. Therefore, induction CYP2C19 by alcohol might affect the *H. pylori* eradication rate.

Furthermore, the female hormone oestrogen has been reported to suppress alcohol decomposition [36]. Alcohol distribution after consumption is also higher in women, which contributes to higher plasma alcohol levels [37]. In addition, body fat is more abundant in women in comparison to men [38], and because alcohol is not readily absorbed into fat tissue due to low fat solubility of alcohol, these factors also contribute to higher plasma alcohol concentration in women in comparison with men [39]. These results suggest that alcohol is likely to exert greater influence in women, and thus largely affects *H. pylori* eradication.

There were four major limitations in this study. First, the frequency of drinking was self-reported by patients, but the volume consumed each time was not tracked. Nonetheless, the National Health and Nutrition Survey in Japan in 2015 shows cross-tabulation of drinking frequency and volumes of daily consumption, and volumes tended to be larger in people who consumed alcohol more often. Second, there were limitations in terms of cases of poor compliance with taking medication. The importance of compliance had been explained carefully to each patient by the pharmacist, duly informing them of the high risk of failure in eradication if medication is skipped. Third, the body mass index (BMI) was not tracked, so it was not taken into consideration in the analysis. BMI is an important risk factor for disease occurrence and has been shown to affect the sexes differentially [40]. Fourth, the types of alcohol consumed were not tracked in this study. Alcohol consumption in Spain, which is mainly in the form of wine, has been found to be associated with successful eradication in a previous study [41]. Beer and rice wine (sake) are the preferred types of alcohol in Japan, but there is a possibility that the type of alcohol may be a factor that affects rate of eradication. This must be discussed further in future studies.

## Conclusions

Collaboration and sharing patient information between a pharmacy and nearby clinic helped to reveal new findings. This collaborative study has shown that a regular drinking habit may lead to failure of primary *H. pylori* eradication in women. Frequent consumption of alcohol is not only likely to affect eradication, but also has a large impact on the bodies of women [42] who are more susceptible than men to the effects of alcohol. Thus, women should take greater care in alcohol consumption.
